# The Influence of Television on Children and Adolescents in an Urban Slum

**DOI:** 10.4103/0970-0218.69291

**Published:** 2010-07

**Authors:** Pankaja Raghav, Alok Kumar

**Affiliations:** Department of Community Medicine, ACS Medical College, Velappan Chawadi, Poonamalle Highway, Chennai–600 077, India. E-mail: drpankajaraghav@gmail.com

**Figure 1:**
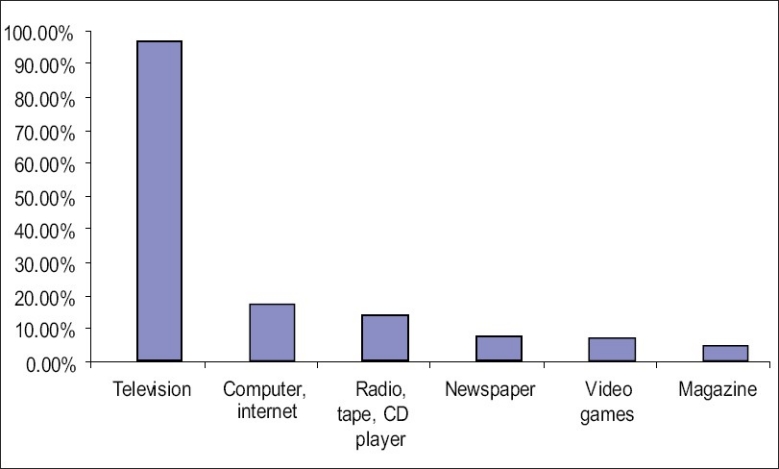
Different media accessed by the children

Sir,

When television first came to India on September 15, 1959 was named as “Doordarshan”(DD) the national television network of India, nobody had ever thought that within a span of less than 50 years, it would cover more than 70 million homes giving a viewing population of 480 million. Television constitutes an important medium widely used to disseminate information to its viewers. It has the unique feature of combining audio and visual technology and serves multiple purposes of entertainment, information, and education. There is no doubt that television has a great influence on children from a very early age itself and it does have an effect on children’s cognitive and social development.(1) Television has the potential to generate both positive and negative effects. An individual child’s developmental level is a critical factor in determining whether the medium will have positive or negative effects.(2) Excessive TV viewing contributes to an increased incidence of obesity and has deleterious effects on learning and academic performance.(3) Time spent with various media may displace other more active and meaningful pursuits such as reading, exercising, or playing with friends. Childhood and adolescence is a time of opportunity and risk. As children and adolescents are the most vulnerable section of the society, the study was carried out on this vulnerable section of the society in an urban slum. The results of the study are interesting. It is observed that [Figure 1] multiple media are accessed by the children and television is the most preferred medium (96.31%). The mean age at which children began watching television was 2.96 years.

Mean hours for which children watched television was 3.56 h.Time spent on television by girls was more (mean=3.73 h) as compared to boys (mean=3.47 h). The reason for this might be non-enrollment of these slum girls in school [Table 1].

**Table 1:** Relationship of gender and the amount of time spent on television

Gender	No.	Mean hours	SD	Range	*t*-value	*P* value
Boys	166	3.47	1.92	0.5–12	1.033	0.303
Girls	95	3.73	2.02	1–10	-	-

Jordan *et al*. in their study observed that most of the children reported spending approximately 3 h per day watching television.(4) Burdette *et al*. in their study found that children watched TV for a mean of 2.2±1.2 h per day.(5) It was observed that 12.26% of the children and adolescents had television in their rooms. Mean hours for which children watched television was more (3.81 h) in children who had television in their rooms as compared to children who did not have television in their rooms (3.51 h). From the research statistics, it can be strongly recommended that parents can help their children make better use of television by scheduling media/television times, limiting children’s total screen time, and helping children and adolescents choose the program appropriate for their age and interests.
